# Maternal factors during pregnancy influencing maternal, fetal, and childhood outcomes

**DOI:** 10.1186/s12916-022-02632-6

**Published:** 2022-11-01

**Authors:** Louis J. Muglia, Katrien Benhalima, Stephen Tong, Susan Ozanne

**Affiliations:** 1 Burroughs Wellcome Fund, Research Triangle Park, Durham, NC USA; 2 Cincinnati Children’s Hospital Medical Center, Department of Pediatrics, University of Cincinnati College of Medicine, Cincinnati, OH USA; 3 University Hospital Gasthuisberg, KU Leuven, Leuven, Belgium; 4 University of Melbourne, Melbourne, Australia; 5 Mercy Perinatal, Heidelberg, Australia; 6 University of Cambridge, Cambridge, UK

## Abstract

Enhancing pregnancy health is known to improve the mother’s and offspring’s life-long well-being. The maternal environment, encompassing genetic factors, impacts of social determinants, the nutritional/metabolic milieu, and infections and inflammation, have immediate consequences for the in utero development of the fetus and long-term programming into childhood and adulthood. Moreover, adverse pregnancy outcomes such as preterm birth or preeclampsia, often attributed to the maternal environmental factors listed above, have been associated with poor maternal cardiometabolic health after pregnancy. In this *BMC Medicine* article collection, we explore a broad spectrum of maternal characteristics across pregnancy and postnatal phenotypes, anticipating substantial cross-fertilization of new understanding and shared mechanisms around diverse outcomes. Advances in the ability to leverage ‘omics across different platforms (genome, transcriptome, proteome, metabolome, microbiome, lipidome), large high-dimensional population databases, and unique cohorts are generating exciting new insights: The first articles in this collection highlight the role of placental biomarkers of preterm birth, metabolic influences on fetal and childhood growth, and the impact of common pre-existing maternal disorders, obesity and smoking on pregnancy outcomes, and the child’s health. As the collection grows, we look forward to seeing the connections emerge across maternal, fetal, and childhood outcomes that will foster new insights and preventative strategies for women.

## Background

The extraordinary, foundational months of pregnancy are a time of emergence of a new life for the conceptus and remarkable physiological and psychological adaptation by the mother. The orchestration of mutual communication between the mother and fetus is the driver of long-term health. It is shaped primarily by the maternal environment, with its genetic, physiologic, nutritional, inflammatory/infection, and psychological components. It has been repeatedly recognized that the in utero environment programs the fetus for lifelong health—the Barker Hypothesis—and pregnancy complications such as preeclampsia and preterm birth impact maternal cardiovascular health [1, 2].

This special collection of articles by *BMC Medicine* seeks to synthesize information related to maternal and offspring outcomes associated with in utero exposures across pregnancy phenotypes and complications. Among the most common maternal traits that impact multiple aspects of fetal outcomes are maternal undernutrition and, more often, maternal overnutrition/obesity, associated with complications from development in an obesogenic environment and influences of gestational diabetes mellitus. In addition, the mechanisms leading to abnormalities in gestational duration and an increased risk for adverse outcomes such as preterm birth are central research targets [3]. The growing opportunity to interrogate “big data” with artificial intelligence or machine learning tools will accelerate this research and help to determine pregnancy interventions that are both safe and effective [4–7].

The editors believe that providing novel insights on exposures and outcomes across pregnancy phenotypes will be mutually informative as many driving determinants are shared. In this editorial, we will highlight some initial contributions to this collection and the new information that has been revealed.

## Towards better health of mother and child—novel insights and potential pathways for intervention

### Revealing underlying mechanisms in preterm birth and potential links to polycystic ovary syndrome

Adverse pregnancy outcomes are common and have generally been refractory to interventions designed to reduce their incidence. Of all obstetric complications, preterm birth towers above nearly all others as the most severe. Affecting 8–10% of all pregnancies, it is depressingly common and can leave the newborn with a lifelong legacy of health deficits [8]: from subtle decrements in developmental outcomes for those born “late preterm” to profound disabilities for those born “extremely preterm” (cerebral palsy, chronic lung conditions, major learning problems) [9].

Spontaneous idiopathic preterm birth has been among the greatest challenges. Until now, there are no generally effective therapeutic interventions, and predictive biomarkers, while beginning to emerge, are limited. The lack of mechanistic insight has resulted in preterm birth being a long-standing leading cause of infant mortality and mortality in children under 5 years of age [10]. However, research of the underlying mechanisms in preterm birth has been greatly accelerated by using hypothesis-free interrogation of large data sets across ‘omics platforms and medical record information using advanced bioinformatic strategies [4–7].

As part of this collection, Tiensuu and colleagues [11] present new data for a candidate biomarker for preterm birth that may also help unravel the underlying mechanisms and is a potential target for interventions. In this study, the investigators evaluated whether placental proteins associated with spontaneous preterm birth can be identified using proteomics. Intriguingly, protein and mRNA levels of alpha-1 antitrypsin (AAT)/SERPINA1 were found to be downregulated on both the maternal and fetal sides of the placenta. This finding served as a basis to investigate whether damaging genetic DNA variations in AAT were also associated with spontaneous preterm birth through whole exome sequencing—and indeed, they were. After revealing this association, the authors performed functional studies, indicating that the downregulation of AAT affects the actin cytoskeletal pathways and extracellular matrix organization.

Beyond identifying relevant biomarkers, there is a strong need for interventions to prevent adverse pregnancy outcomes. The Tiensuu study moves forward with one strong candidate for such an intervention. Moreover, their approach of utilizing multiple association strategies to provide further evidence for a particular finding can be applied across various disease phenotypes.

Major risk factors for preterm birth have long been elucidated [8, 9], such as prior preterm birth, early rupture of membranes, or co-existing medical conditions such as polycystic ovary syndrome (PCOS), as reported by others in this special collection [12]. Rocha and colleagues address an interesting question: for those in their second pregnancy and birth preterm, do risk factors associated with their preterm birth differ depending on whether or not their first infant was born preterm?

To address this question, the authors examined a large retrospective dataset from Brazil representing 1.7 million births [13]. They focused on women who had a preterm birth in their second pregnancy and split them according to whether their first pregnancy was delivered at full term (> 37 weeks gestation, “incident preterm birth” cohort) or they previously had a preterm birth (< 37 weeks gestation, “recurrent preterm birth” cohort).

Interestingly, the incident but not the recurrent preterm birth cohort had significant associations with household overcrowding, variations in ethnicity (being black, mixed-race, or indigenous had raised risks), being a younger mother (14–19 years), and having had a prior cesarean section, with odds ratios ranging from 1.04 to 1.34. Both cohorts were associated with reduced prenatal visits with higher odds ratios in the incidence preterm birth cohort. Notably, many of these risk factors likely reflect socioeconomic deprivation, stress, low educational attainment, and smoking—established risk factors for preterm birth [8].

Surprisingly, in both cohorts, being single or a widow conferred a 10–15% reduced risk of preterm birth compared to those who were married or in a civil union. While interesting, this finding is difficult to explain, and we do not suggest encouraging women to be single is a promising public health strategy to reduce preterm birth rates.

In another contribution to this collection, Subramanian and colleagues [12] present data indicating a convincing link between preterm birth and PCOS, a condition affecting 10% of women. While defined by a varied constellation of signs and symptoms—cysts on the ovary, biochemical or clinical evidence of androgen excess, oligo/anovulation [14]—PCOS is, at its heart, a metabolic disorder [15]. As a chronic condition that never retreats, those affected incur the risk of developing metabolic-related conditions as they age, especially diabetes and obesity [15].

Given PCOS is the most common endocrine disorder among women of reproductive age, it will invariably intersect with many pregnancies. In their retrospective study, Subramanian et al. examined the link between a pre-specified set of serious obstetric complications, including preterm birth, fetal size, mode of birth and stillbirth, and PCOS based on just under 140,000 pregnancies in the UK, of which 27,586 were affected by PCOS. While the lift in preterm birth risk in women affected by PCOS was modest (an 11% relative rise on the adjusted odds ratio), it could be substantiated by further sub-analyses. These findings concur with a recent study in a Swedish population, indicating an apparent doubling in the risk of extreme preterm birth < 28 weeks gestation in women suffering from PCOS and, thus, an even larger effect size [16].

But how is the link between PCOS and preterm birth explained? The authors muse over potential causes such as a shared genetic polymorphism between preterm birth and PCOS or a dysregulated hypothalamic-pituitary-adrenal axis tipping off a biological cascade that ends in spontaneous preterm birth. However, as the team did not adjust for important pregnancy-induced complications strongly associated with both PCOS and preterm birth (such as gestational diabetes and hypertensive disorders), more likely, the presence of such complications led to the excess in preterm births. They also found PCOS associated with a modestly increased risk of a cesarean section but no apparent link with stillbirth. While this finding seems reassuring, the study was likely underpowered to explore this outcome.

Finally, there may be a fascinating biological message buried within their apparently plain finding that PCOS is not associated with the birth of babies that are either small or large for gestational age. It suggests placental function may be surprisingly resistant to the multiple metabolic perturbations occurring within the mother, which would be a reassuring finding.

### The impact of environmental exposures—metabolic in utero environment, obesity, and smoking during pregnancy

In addition to adverse obstetric outcomes such as preterm birth and associated risk factors, obesity during pregnancy is of great concern. Obesity rates continue to increase across the globe in all age groups in the population, including women of childbearing age [17]. Consequently, in a growing number of countries, over half of the pregnant women are now either obese or overweight.

Obesity is associated with immediate detrimental consequences for the mother and baby, including increased risk of gestational diabetes, preeclampsia, and the need for a caesarian section [18]. In addition, it is established that children born to obese women are at increased risk of becoming obese and developing type 2 diabetes and cardiovascular diseases. Furthermore, evidence suggests that at least part of this transmission of poor cardio-metabolic health from mother to child is driven by non-genetic factors. Notably, the latter provides an opportunity for early intervention before disease pathology is established [19].

Currently, it is not known which children born to obese mothers will follow a higher-than-normal body mass index growth trajectory and become overweight and ultimately obese. In this article collection, Gomes and colleagues address this knowledge deficit using the mother-child cohort study Programming of Enhanced Adiposity Risk in Childhood–Early Screening (PEACHES), which comprised 1671 mothers with pre-conception obesity and without (controls) and their offspring. They identified a “high-risk” subpopulation of offspring born to obese mothers susceptible to early upper deviations from healthy weight gain trajectories present within the first few months of life and progressing to overweight/obesity by 5 years of age. Hence, they established a novel sequential prediction system to allow early-risk stratification and re-evaluation to prevent a “higher-than-normal BMI growth pattern” at a subclinical stage preceding overweight. Maternal smoking and excessive gestational weight gain were the strongest predictors of a higher-than-normal BMI growth pattern by 3 months of age. Importantly, they validated these findings in the independent Perinatal Prevention of Obesity (PEPO) cohort, comprising 11,730 mother-child pairs recruited around 6 years of age. These findings take us a step closer to developing cost-effective and personalized advice and measures, counteracting the risk of early excess weight gain and ultimately preventing childhood obesity.

Several articles in this collection have focused on the metabolic environment in utero and the impact of environmental exposures in pregnancy on the mother’s and offspring’s long-term metabolic health. For example, the large mother-offspring Asian cohort study Growing Up in Singapore Towards healthy Outcomes (GUSTO), consisting of 1247 women from Singapore, studied the changes of 480 lipid species in the plasma of women during pregnancy (antenatal vs postnatal) and their offspring during development (cord blood and 6-year-old child plasma) [20]. This study demonstrated that around 36% of the profiled lipids increased in circulation during pregnancy, with phosphatidylethanolamine levels changing the most compared to pre-pregnancy. Compared to the gestating mother, the cord blood showed a lower concentration of most lipids, and a higher concentration of lysophospholipids, suggesting the specific developmental needs of the growing fetus. Pre-pregnancy BMI was specifically associated with a decrease in the levels of phospholipids, sphingomyelin, and several triacylglycerol species in pregnancy, highlighting the importance of managing obesity before pregnancy. Notably, lipid species associated with the child’s BMI were very similar to those associated with the adult’s BMI. This overlapping effect of adiposity, as observed in 6-year-old children and postnatal mothers in this study, may be influenced by the similarities in the diet apart from other factors such as genetics and shared lifestyle. The findings of this study were validated in an independent Caucasian birth cohort and provide an important resource for future research targeting early nutritional interventions to benefit maternal and child metabolic health.

Also focusing on the influencing factors on metabolic health, a Swedish nationwide register-based study investigated the association between maternal smoking during pregnancy and type 1 diabetes in the offspring [21]. The cohort consisted of nearly three million children born between the years 1983 and 2014, with follow-up until 2020. In addition, a nested case-control study was performed comparing children with type 1 diabetes to their age-matched siblings. A total of 18,617 children developed type 1 diabetes. Interestingly, maternal smoking during pregnancy was associated with a 22% lower risk of offspring type 1 diabetes in the full cohort. Although these data suggest a protective effect of maternal smoking on the risk for offspring to develop type 1 diabetes, mechanistic studies are needed to elucidate the underlying pathways behind this link. Despite these findings, we emphasize that smoking during pregnancy should be strongly advised against since it has severe effects on fetal and childhood health [21].

For example, a longitudinal study by Howell and colleagues in this collection provides evidence that maternal smoking is also associated with shorter offspring telomere length [22]. Acting as a mitotic clock to the cell, these hexameric repeat sequences found at the ends of chromosomes shorten with cell division [23] and, as shown recently, as a consequence of oxidative damage. Therefore, they represent good biomarkers of cellular aging and also exposure to oxidative damage [23]. Accelerated aging has been suggested as one potential mechanism linking suboptimal in utero exposures to long-term health. However, most evidence has primarily come from studies of suboptimal in utero nutritional exposures [24].

Howell et al. demonstrated that maternal prenatal smoking predicted greater telomere shortening by measuring the telomere length in buccal cells in infants from 4 to 18 months of age. They also showed that the relationship between maternal prenatal smoking and offspring attention-deficit/hyperactivity disorder (ADHD) was modulated by telomere length. Paradoxically, ADHD was associated with less telomere attrition for children whose mothers smoked. It is not known if these differences in buccal cell telomere length are reflective of the differences in other cell types, such as those in the brain. However, if similar differences are also present in brain tissue, this finding could indicate delayed maturation of cortical cells, which could provide a mechanistic link to ADHD.

## Conclusions

As demonstrated by the initial series of articles published in this collection, the ability to utilize now more refined technologies to elucidate the underlying mechanisms that drive adverse pregnancy outcomes, such as preterm birth and metabolic risks for both the mother and fetus, has revealed new insights and potential pathways for intervention. Moreover, a deeper understanding of how these diverse outcomes are influenced by maternal co-morbidities such as maternal PCOS or smoking status is emerging.

However, to have a real impact on public health, these robust, reliable data and their implications need to be implemented in physician practice and be used for therapy development for a historically under-explored and invested group—pregnant women.

It will be essential to figure out how these findings can be used to tackle challenges related to lifestyle factors such as maternal obesity or smoking that have been refractory to preventive strategies and interventions. As the recognition of these influencing factors on maternal, fetal, and childhood outcomes across the lifespan emerges, we are encouraged that it will ultimately benefit the mother’s and child’s health.

We have enjoyed learning from this initial set of articles and look forward to future contributions to this collection.

## Data Availability

All data discussed in this editorial are included in this published article.
